# Supplementary material for: Lychee Fermented by Mixed Probiotic Strains Alleviates D-Galactose-Induced Skeletal Muscle and Intestinal Aging in Mice
Source: Foods. 2025 Oct 29;14(21):3684. doi: 10.3390/foods14213684 (PMC12609573; doi:10.3390/foods14213684)
Supplement: Supplementary file 1 [file foods-14-03684-s001.zip › foods-3922285-supplementary.pdf]

Supporting Information

Lychee Fermented by Mixed Probiotic Strains Alleviates D-Galactose-Induced Skeletal Muscle and Intestinal Aging in Mice

Tables

Table S1. Primers used for Real-time qPCR

Gene	5’→3’	3’→5’
β-Actin	CCTAGAAGCATTTGCGGTGCACGATG	TCATGAAGTGTGACGTTGACATCCGT
p16	CGAGGACCCCACTACCTTCT	GATGTCTTGATGTCCCCGCT
p21	GCAGATCCACAGCGATATCCA	AACAGGTCGACATCACCAG
Il-1β	GAAATGCCACCTTTTGACAGTG	TGGATGCTCTCATCAGGACAG
Il-6	TAGTCCTTCCTACCCCAATTTCC	TTGGTCCTTAGCCACTCCTTC
Tnf-α	CCCTCACACTCAGATCATCTTCT	GCTACGACGTGGGCTACAG
Mcp1	TTAAAAACCTGGATCGGAACCAA	GCATTAGCTTCAGATTTACGGGT
Cxcl1	TCCAGAGCTTGAAGGTGTTGCC	AACCAAGGGAGCTTCAGGGTCA
ZO-1	GTTGGTACGGTGCCCTGAAAGA	GCTGACAGGTAGGACAGACGAT
Claudin-1	TCACTTTTCCTGCGGTGACTT	CCAATGTCAATGGCAACACCC
Occludin	TCACTTTTCCTGCGGTGACTT	GGGAACGTGGCCGATATAAT
MUC2	TGCTGACGAGTGGTTGGTGAAT	GATGAGGTGGCAGACAGGAGAC

Table S2. Statistics of Sequence Information After Denoising

Group_info	Sample_info	ASVs_num	Seq_num
Control	C1	721	32571
	C2	740	33577
	C3	601	31258
	C4	618	28875
	C5	521	36760
	C6	384	39053
D-gal	D1	488	31396
	D2	512	32772
	D3	474	29104
	D4	532	28171
	D5	484	31309
	D6	472	25354
HLF	L1	613	33654
	L2	641	32450
	L3	458	31842
	L4	554	29523
	L5	600	33925
	L6	1008	33765

Table S3. The representative differential metabolites in LF vs LJ group

Compounds	Formula	VIP	Fold Change after fermentation	Type
Asiaticoside	C48H78O19	1.15	184.32	Up
L-(-)-3-Phenyllactic acid	C9H10O3	1.14	172.80	Up
n-Oleoylethanolamine	C20H39NO2	1.14	123.40	Up
2-Hydroxy-4-methylvaleric acid	C6H12O3	1.15	123.02	Up
2-Isopropylmalic acid	C7H12O5	1.00	121.08	Up
Sphingosine	C18H37NO2	1.15	112.78	Up
3-Hydroxybutyric acid	C4H8O3	1.15	95.50	Up

Gulonic acid	C6H12O7	1.15	87.73	Up
N-Acetyltyramine	C10H13NO2	1.15	61.32	Up
3-Hydroxysuberic acid	C8H14O5	1.12	54.93	Up
L-Histidinol	C6H11N3O	1.00	48.62	Up
Ethyl caffeate	C11H12O4	1.15	30.76	Up
1-Acetylproline	C7H11NO3	1.15	29.47	Up
O-Acetylcarnitine	[C9H18NO4]+	1.15	23.79	Up
Gluconic acid	C6H12O7	1.02	23.30	Up
Citramalic acid	C5H8O5	1.15	20.18	Up
Propionic acid	C3H6O2	1.15	19.27	Up
Thiamine	C12H17N4OS	1.15	18.38	Up
Benzoylformic acid	C8H6O3	1.15	17.49	Up
Fraxetin	C10H8O5	1.14	16.51	Up
Methyl 2-amino-3-phenylpropanoate	C10H13NO2	1.15	16.10	Up
Daphnetin	C9H6O4	1.14	14.39	Up
Naringenin chalcone	C15H12O5	1.15	13.58	Up
Isorhamnetin	C16H12O7	1.15	11.78	Up
Ethyl glucuronide	C8H14O7	1.15	11.03	Up
Ilicic acid	C15H24O3	1.14	10.89	Up
Succinic acid	C4H6O4	1.15	10.79	Up
Palmitoleic acid	C16H30O2	1.14	10.73	Up
Indole-3-lactic acid	C11H11NO3	1.15	10.69	Up
3-Hydroxypentanoic acid	C5H10O3	1.04	10.19	Up
Kojic acid	C6H6O4	1.14	9.65	Up
3,4-Dihydroxybenzaldehyde	C7H6O3	1.11	9.56	Up
2-Phenylacetamide	C8H9NO	1.12	9.55	Up
2-Hydroxy-3,4-dimethoxybenzoic acid	C9H10O5	1.14	9.33	Up
3-(3-Hydroxy-phenyl)-acrylic acid	C9H8O3	1.14	9.23	Up
Cyanidin	C15H11O6	1.15	8.89	Up
Momordin Ic	C41H64O13	1.15	8.45	Up
4-Hydroxyquinoline	C9H7NO	1.15	7.67	Up
Eriodictyol	C15H12O6	1.14	7.39	Up
4-Hydroxyisoleucine	C6H13NO3	1.14	7.11	Up
3-p-Coumaroylquinic acid	C16H18O8	1.12	7.00	Up
Catechol	C6H6O2	1.13	6.88	Up
Vanillic acid	C8H8O4	1.15	6.86	Up
(-)-Epicatechin	C15H14O6	1.13	6.74	Up
Neobavaisoflavone	C20H18O4	1.15	6.57	Up
2,5-Dihydroxybenzoic acid	C7H6O4	1.00	6.19	Up
2,3-Dihydroxybenzoic acid	C7H6O4	1.12	4.83	Up
Gallic acid	C7H6O5	1.12	4.67	Up
3-Methylbenzoate	C8H8O2	1.15	4.45	Up
Shanzhiside methyl ester	C17H26O11	1.00	4.05	Up
Pantothenic acid	C9H17NO5	1.15	4.00	Up
Ethyl ferulate	C12H14O4	1.12	3.99	Up
Maltol	C6H6O3	1.00	3.92	Up
Pinocembrine	C15H12O4	1.14	3.81	Up
3-(4-Hydroxyphenyl)-2-propenoic	C9H8O3	1.08	3.71	Up

acid				
2-Hydroxy-3-methylbutyric acid	C5H10O3	1.05	3.67	Up
Vernolic acid	C18H32O3	1.14	3.67	Up
Diosmetin-7-O-neohesperidoside	C28H32O15	1.14	3.61	Up
2-Hydroxyisobutyric acid	C4H8O3	1.12	3.44	Up
Taxifolin	C15H12O7	1.15	3.43	Up
Phenylacetaldehyde	C8H8O	1.12	3.42	Up
Cinchonidine	C19H22N2O	1.13	3.39	Up
12-Hydroxyjasmonic acid	C12H18O4	1.14	3.34	Up
Fraxidin	C11H10O5	1.14	3.17	Up
Pyrrole-2-carboxylic acid	C5H5NO2	1.14	3.04	Up
3-(3-Methoxyphenyl)propionic acid	C10H12O3	1.14	3.00	Up
Isophorone	C9H14O	1.11	2.92	Up
3-Hydroxynonanoic acid	C9H18O3	1.12	2.89	Up
4-Hydroxycinnamic acid	C9H8O3	1.13	2.84	Up
Shikimic acid	C7H10O5	1.15	2.80	Up
p-Coumaryl alcohol	C9H10O2	1.11	2.79	Up
Cinnamaldehyde	C9H8O	1.11	2.74	Up
Enoxolone	C30H46O4	1.05	2.68	Up
4-Methoxycinnamic acid	C10H10O3	1.12	2.68	Up
5,7-Dihydroxyflavanone	C15H12O4	1.15	2.66	Up
Perillene	C10H14O	1.15	2.65	Up
5-O-Feruloylquinic acid	C17H20O9	1.09	2.63	Up
Inosine	C10H12N4O5	1.10	2.61	Up
Fisetin	C15H10O6	1.12	2.59	Up
Phloretin	C15H14O5	1.12	2.58	Up
Caffeic acid	C9H8O4	1.11	2.50	Up
2-Methoxybenzoic acid	C8H8O3	1.13	2.31	Up
Kaempferol	C15H10O6	1.14	2.29	Up
Quinic acid	C7H12O6	1.14	2.28	Up
5,7-Dihydroxy-2-(4-hydroxyphenyl)chroman-4-one	C15H12O5	1.14	2.20	Up
Scopoletin		1.09	2.14	Up
Phenylpyruvic acid	C9H8O3	1.10	2.14	Up
8-Prenylnaringenin	C20H20O5	1.13	2.09	Up
Naringenin	C15H12O5	1.10	2.09	Up
Salicin	C13H18O7	1.10	2.09	Up
Kaempferol-3-Glucoside-3"-Rhamnoside	C27H30O15	1.10	2.09	Up
Phenyl acetate	C8H8O2	1.14	2.02	Up
Salicylic acid glucoside	C13H16O8	1.00	2.02	Up
6,7-Dimethoxy-8-(beta-D-glucopyranosyloxy)-2H-1-benzopyran-2-one	C17H20O10	1.13	1.89	Up
p-Mentha-1-ene-8-yl 6-O-(alpha-L-rhamnopyranosyl)-beta-D-glucopyranoside	C22H38O10	0.98	1.82	Up
Cyanidin 3-O-glucoside	[C21H21O11]+	1.14	1.79	Up
4-Hydroxybenzoic acid 4-O-glucoside	C13H16O8	1.00	1.79	Up
Luteolin 7-O-rutinoside	C27H30O15	1.09	1.73	Up

Apigenin 7-O-(2G-rhamnosyl)gentiobioside	C33H40O19	1.09	1.71	Up
Mundulone	C26H26O6	1.13	1.71	Up
Lyoniside	C27H36O12	1.10	1.70	Up
3,4-Dihydro-6,8-dihydroxy-3-(10-hydroxyundecyl)isocoumarin	C20H30O5	1.00	1.69	Up
Curcumol	C15H24O2	1.12	1.67	Up
4-Pyridoxic acid	C8H9NO4	1.00	1.65	Up
Naringin	C27H32O14	1.11	1.64	Up
Dioctylamine	C16H35N	1.09	1.61	Up
Kaempferol 3-O-[alpha-L-rhamnopyranosyl(1->2)-beta-D-glucopyranosyl]-7-O-alpha-L-rhamnopyranoside	C33H40O19	1.15	1.56	Up
Vanillin	C8H8O3	1.12	1.50	Up
Luteolin	C15H10O6	1.03	1.50	Up
Isoquercitrin	C21H20O12	1.01	1.50	Up
Quercetin	C15H10O7	1.14	-1.50	Down
Kaempferol 7-O-rhamnoside	C21H20O10	1.11	-1.66	Down
Kaempferol 3,7-diglucoside	C27H30O16	1.00	-1.67	Down
(+)-Gallocatechin		1.14	-1.69	Down
Scopolin	C16H18O9	1.15	-2.54	Down
4-Hydroxybenzaldehyde	C7H6O2	1.15	-2.69	Down
Proanthocyanidin A2	C30H24O12	1.15	-3.59	Down
Coumaric acid 4-O-glucoside	C15H18O8	1.14	-3.74	Down
2,3-Butanedione	C4H6O2	1.15	-3.82	Down
Galactaric acid	C6H10O8	1.14	-3.83	Down
Kaempferol 7-neohesperidoside	C27H30O15	1.10	-4.27	Down
Peonidin-3-O-alpha-arabinoside	C21H20O10	1.15	-4.52	Down
Procyanidin B2	C30H26O12	1.15	-4.54	Down
Procyanidin C1	C45H38O18	1.08	-6.45	Down
Fructose (Generic Ketohexose)	C6H12O6	1.15	-7.79	Down
Cryptochlorogenic acid	C16H18O9	1.00	-8.10	Down
prim-O-b-D-Glucosylcimifugin	C22H28O11	1.15	-8.22	Down
D-Glucose	C6H12O6	1.01	-8.94	Down
Procyanidin B1	C30H26O12	1.15	-9.40	Down
Petunidin 3-galactoside	C22H23O12	1.08	-9.84	Down
Raffinose	C18H32O16	1.15	-9.85	Down
Isorhamnetin 3-galactoside	C22H22O12	1.15	-13.21	Down
Fraxin	C16H18O10	1.11	-13.36	Down
3,4-Dihydroxybenzoic acid	C7H6O4	1.15	-13.97	Down
beta-Gentiobiose	C12H22O11	1.15	-14.40	Down
Tryptophan	C11H12N2O2	1.15	-18.30	Down
Glutamine	C5H10N2O3	1.15	-25.26	Down
Aspartic acid	C4H7NO4	1.15	-48.06	Down
Trehalose	C12H22O11	1.00	-65.06	Down
Sucrose	C12H22O11	1.00	-82.97	Down

Figures

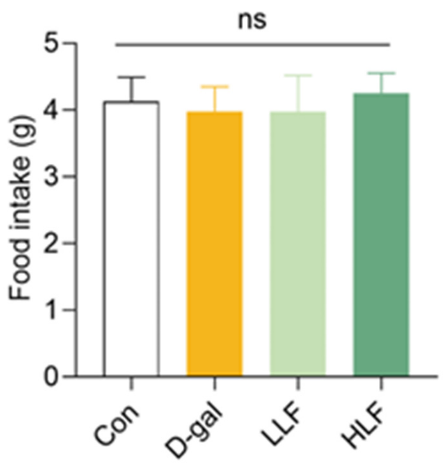

Figure S1. Average daily food intake of mice in each group during LF intervention.

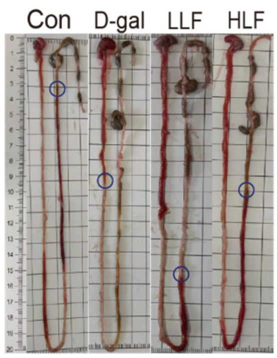

Figure S2. Advance distance of carmine dye in the small intestine of different groups.

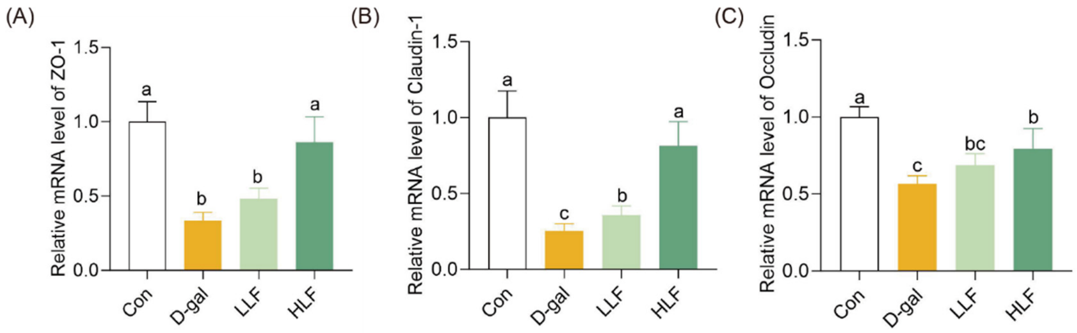

Figure S3. Relative mRNA expression levels of tight junction proteins ZO-1, Claudin-1, and Occludin in colon tissues in colon tissues

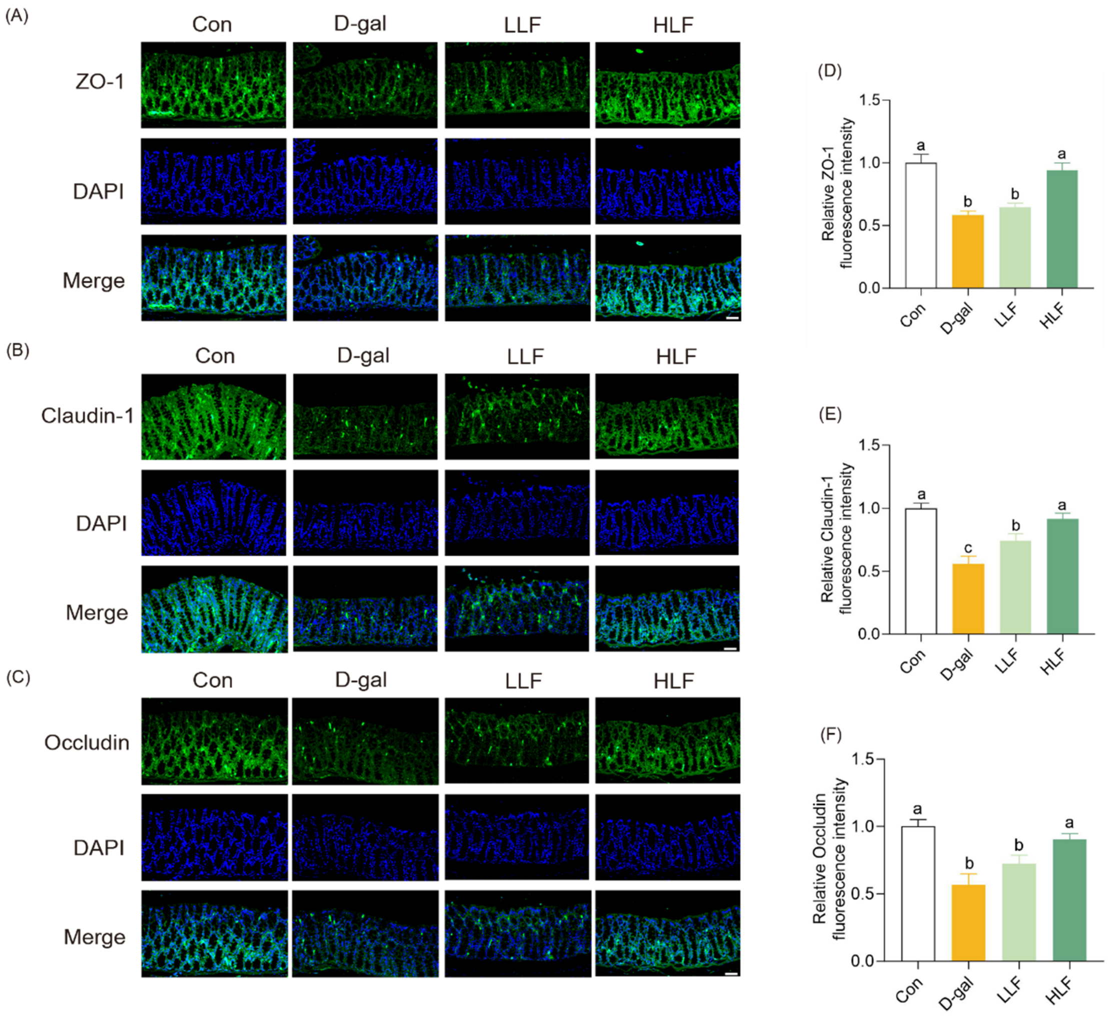

Figure S4. Staining of ZO-1, Claudin-1 and Occludin (green) in the colon from different groups, with representative images and relative fluorescence intensity. Scale bars = 100 μm. The letters above the bars indicate statistical differences ($P < 0.05$).

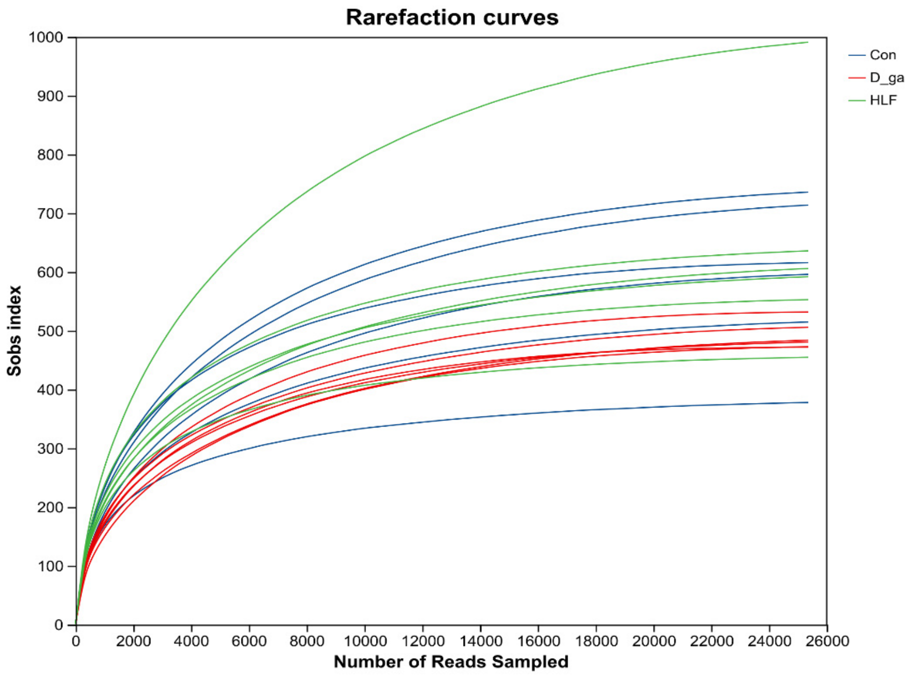

Figure S5. Rarefaction analysis of partial 16S rRNA gene sequence to estimate the microbial diversity of each sample.

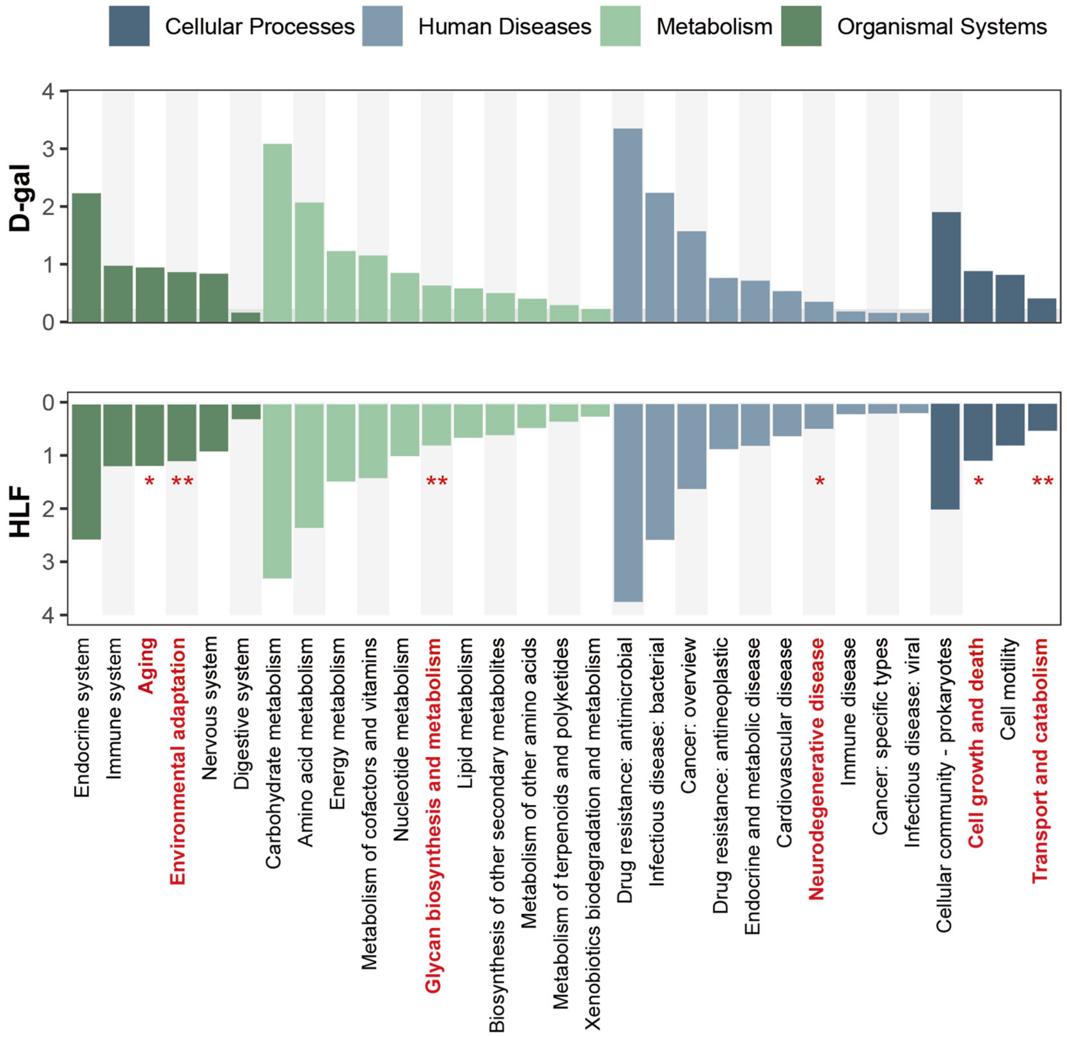

Figure S6. Differential enrichment analysis of microbiota in the KEGG pathways between the D-gal and HLF groups.

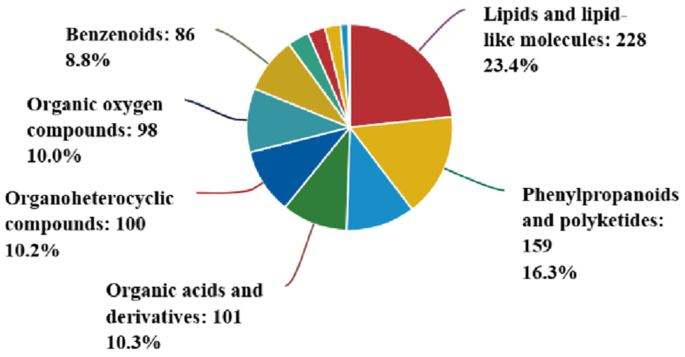

Figure S7. Category of all detected metabolites in all samples

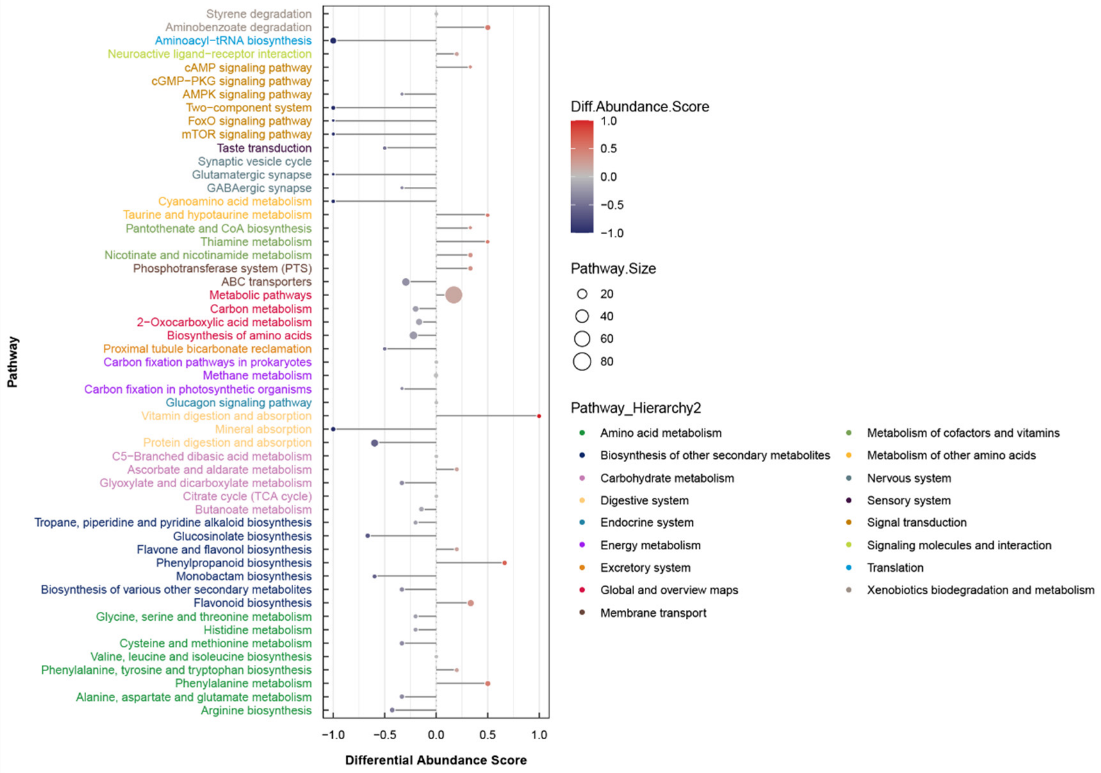

Figure S8. The metabolites enriched into several metabolic pathways by KEGG enrichment analysis
